# Effect of Hydrostatic Pressure Upon the Relaxation of Birefringence in Amorphous Solids

**DOI:** 10.6028/jres.065A.033

**Published:** 1961-08-01

**Authors:** Roy M. Waxler, Leason H. Adams

## Abstract

A study was made of the effect of hydrostatic pressure upon the relaxation of birefringence in two commercial plastics which were glasslike in nature. The birefringence was introduced into the materials by loading in uniaxial compression, and the decay of birefringence with time was measured using a polarimeter. The rate at which the optical path-difference disappeared was greatly inhibited by the hydrostatic pressure, and a pressure of 10,000 bars was found to be sufficient to stop the release completely. The results are interpreted as the effect of hydrostatic pressure upon the mobility of the materials. Some experiments were conducted to determine the effect of temperature upon the relaxation, that the effects of pressure and temperature might be compared.

## 1. Introduction

When an amorphous solid is subjected to a uniaxial stress, it becomes birefringent, and the subsequent decay of this birefringence is associated with a relaxation of the stress. This phenomenon is attributed to flow in the material. The relaxation of internal stress in glass as a function of temperature was investigated some years ago by Adams and Williamson [1]^1^ in their work on annealing. Investigations by Lillie [2] and Isard and Douglas [3] give evidence that in the annealing of silicate glasses, the release is accomplished essentially by viscous flow. Regardless of the mechanism by which the stress is released, the influence of hydrostatic pressure upon this mobility is of general interest in solid state physics. The hydrostatic pressure of itself does not introduce any double refraction into the material, as will be shown.

## 2. Measurement of Optical Path-Difference

In measuring optical path-difference it is customary to use a Babinet compensator or a graduated quartz wedge, which instruments afford a sensitivity of about ± 5 m*μ*. A polarimeter developed by Goranson and Adams [4] affords a sensitivity of ± 0.05 m*μ*, and the measurements may be made easily.^2^ Plane-polarized light is rotated through an angle equal to one-half the phase lag produced by double refraction in the specimen by this instrument. Since the measurements were of angular displacement, it was found to be convenient and adequate to express the optical path-difference as phase lag in the specimen in degrees, this value being twice the measured value of the rotation. The instrument is equipped with a graduated quartz wedge used in determining the multiples of 360° when the phase lag in the specimen exceeds one whole wavelength. A high pressure mercury arc lamp was used with optical filters to isolate the green line of 5461A.

## 3. Pressure Apparatus

The apparatus and techniques for obtaining high hydrostatic pressures have been described in detail by Weir [5]. Briefly, a heavy-walled pressure vessel has a smooth bore which contains a light petroleum distillate. By means of a hydraulic press, a leak-proof piston is forced into the bore of the vessel thereby compressing the liquid and generating hydrostatic pressure. The apparatus is designed for pressures up to 10,000 bars, and the pressure is measured internally by means of a manganin pressure gage to a precision of ±⅓ bar.

## 4. Choice of Material and Method of Introducing Optical Path Difference

It was convenient to select a glass such as glucose, or polybutyl methacrylate, that would anneal around room temperature. It was planned to introduce double refraction into a specimen by plunging it into a bath at low temperature, and then to observe the relaxation at room temperature. Some polybutyl methacrylate was polymerized and molded into blocks. The dimensions of these blocks were reduced to 1½ × 9/16 × ⅜ in. to permit them to enter the bore of the pressure vessel. The amount of double refraction introduced into one of these small specimens by quenching varied markedly so that it was impossible to find an area with sufficient uniformity to allow a good measurement.

**Figure 1:**
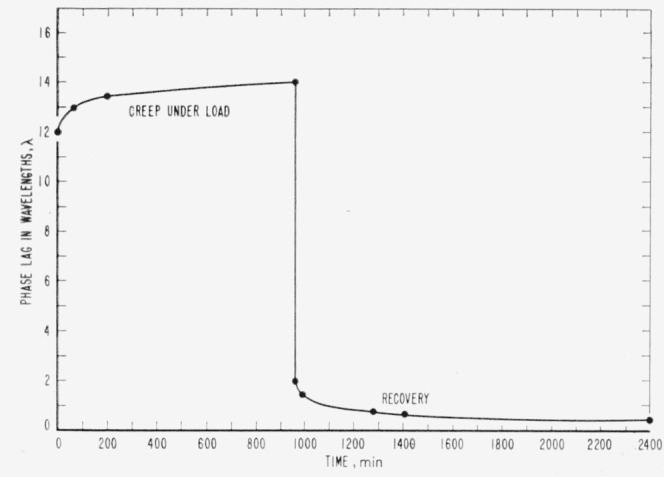
Birefringence in a specimen of allyl diglycol carbonate when loaded to 350 kg/cm^2^ for 960 minutes and then unloaded.

Considering the difficulties with temperature- induced photoelastic effects, a method was sought that would introduce into a specimen a uniform birefringence that would decay with time. It has been found in early work on photoelastic stress analysis [6] that when a transparent plastic is loaded and the load held constant over a period of time, the optical path difference caused by the stress does not remain constant but increases with time. Likewise, when the load is removed, the optical path difference does not go to zero immediately but decays gradually. These effects are shown very roughly for allyl diglycol carbonate in figure 1, where a specimen in the form of a block 1½ × 9/16 × ⅜ in. was loaded lengthwise in uniaxial compression. Allyl diglycol carbonate is widely used in stress analysis, having a stress-optical coefficient of 26.5 brewsters, where 1 brewster=10^−13^ (cm)^2^/dyne. It is available in large clear sheets that give excellent transmission of light in the visible region.

A small loading mechanism which has been described previously [7] was employed to insure a uniform and uniaxial compression of the specimen. This device was modified for the present work, so that instead of using suspended weights, the load was applied by means of a testing machine. A specimen of allyl diglycol carbonate with the same dimensions as used previously was loaded to 350 kg/cm^2^, and the platens of the testing machine were then held in this fixed spatial position for a period of 16 hr. Upon removing the residual load, measurement of the specimen in the polarimeter showed that there was a phase lag of about 720°, or two wavelengths of the 5461 A line. This residual phase lag reduced to about 20 percent of its original value in the course of a day, and when a plot was made of the phase lag in the specimen versus time, the points fell on a smooth curve.

Change in birefringence under load was also observed in commercial polymethyl methacrylate. Like allyl diglycol carbonate, this material is available in large, clear sheets, and affords excellent transmission in the visible region of the spectrum. However, polymethyl methacrylate has a much smaller stress-optical coefficient (about 3.75 brewsters). A specimen of this plastic, with the same dimensions as used previously, was loaded in uniaxial compression to 700 kg/cm^2^, and the platens of the testing machine were then held in this fixed spatial position for a period of 16 hr. Upon removal of the residual load, the specimen exhibited a residual phase lag corresponding to three-quarters of a wavelength of mercury green light, and this lag gradually reduced to about 20 percent of its original value in the course of a day.

For both ally] diglycol carbonate and polymethyl methacrylate, X-ray diffraction patterns obtained from a Geiger counter diffractometer showed the presence of broad peaks typical of amorphous materials.

A piece of allyl diglycol carbonate which showed no evidence of double refraction when examined in polarized light was placed in the pressure bomb and subjected to a pressure of 10,000 bars for a period of 10 min. The pressure was then released, and the specimen, upon removal from the bomb, still showed no sign of double refraction after temperature equilibrium had been established. The result of this experiment was taken as verification of the statement in the introduction that hydrostatic pressure of itself does not introduce any double refraction into an amorphous solid.

Since the relaxation curve for allyl diglycol carbonate was not closely reproducible for specimens treated in the same way, it was decided to load a specimen double the usual length, one half to be placed in the pressure bomb at elevated pressure, and the other half to be used as a reference specimen at atmospheric pressure. Upon removal from the loading apparatus, examination in polarized light showed that the optical path-difference in each half was the same.

## 5. Experimental Procedure

The above approach was followed in an experiment where the pressure bomb was raised to 10,000 bars, this pressure being selected because a large effect was sought. Making allowances for the time spent in raising and lowering the pressure in the bomb, the results of the experiment indicated that there was no relaxation of the specimen at all during the time it was under the pressure of 10,000 bars. This initial experiment was followed by similar ones using pressures of 2,100 bars, 1,000 bars, and 500 bars, and it could be seen, qualitatively, that the relaxation proceeded at a greater rate at each succeedingly lower pressure.

To ascertain that this inhibiting of the relaxation was not unique for allyl diglycol carbonate, two experiments of a similar nature were conducted with polymethyl methacrylate at pressures of 2,000 bars and 1,000 bars. It was necessary to enclose a polymethyl methacrylate specimen in a polyethylene bag in order to protect it from attack by the petroleum fraction used in the pressure bomb. The results of the experiments agreed qualitatively with earlier results, that the relaxation proceeded at a greater rate at lower pressure. Since the relaxation curves for polymethyl methacrylate had been found to be reproducible at atmospheric pressure, it was not necessary to have an individual reference specimen for each test, as with allyl diglycol carbonate.

All these experiments on both plastics were conducted at a temperature of 24 °C.

## 6. Calculation of Relative Mobilities

In studying the relaxation of each amorphous solid, an empirical relationship between the variables was sought for greater convenience in handling the data. It was found that the data could be fitted very well to the equation

$$\frac{d\phi }{dt}=-A\phi^{4}$$ (1)

where *ϕ* represents the phase lag in a specimen, *t* represents the time, and *A* is a constant for a given temperature and pressure. Integrating equation (1) one obtains

$$\frac{1}{\phi^{3}}-\frac{1}{\phi_{0}^{3}}=3At,$$ (2)

**Figure 2:**
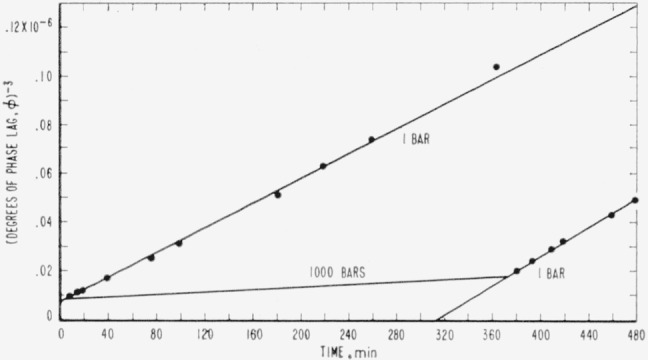
The reciprocal cubed of phase lag in allyl diglycol carbonate as a function of time, showing the effect of a hydrostatic pressure of 1,000 bars in comparison with that of 1 bar, the plot at 1,000 bars being inferred.

*ϕ*_0_ being the initial phase lag in the specimen. It can be seen that by plotting the reciprocal cubed of phase lag versus time that a straight line results. Furthermore, knowing the time and amount of phase lag in the specimen when it was put into the pressure bomb, and the time and amount of phase lag when it was removed, it was possible to infer the rate of the relaxation under pressure. This is shown in figure 2 for allyl diglycol carbonate subjected to a pressure of 1,000 bars.

It was assumed that the constant, 3*A*, was a measure of the tendency to flow in the material, or of its mobility in the most general sense. The ratio of the slopes of the two straight lines shown in figure 2 was taken as an expression of the mobility at elevated pressure relative to the mobility at atmospheric pressure; i.e., the relative mobility was taken as *A_p_/A*_0_ where *3A*_0_ and 3*A_p_* are respectively the slopes at atmospheric pressure and at elevated pressure. The data for each experiment were treated in this manner, and relative mobilities were calculated in each case.

## 7. A New Experimental Method

There were several objections to making measurements in the foregoing manner: (1) Only a rough estimate could be made of the time that a specimen was affected by the pressure, because 10 min would elapse in bringing the bomb up to pressure, and about the same time would elapse in releasing the pressure; (2) it was known that there was a temperature rise due to adiabatic compression, and a lowering of temperature due to adiabatic expansion; (3) one could see that the fourth power relationship for the relaxation (eq 1) held at atmospheric pressure, but there was no assurance that the same law applied while a specimen was under pressure.

These considerations made it desirable to observe the relaxation while a specimen was under pressure. Fortunately, for this purpose, it was possible to utilize a pressure vessel equipped with glass windows 3 [8]. The windows were supported by hardened stainless steel plugs, the contacting faces being ground optically flat. The combination of window and supporting steel plug made a Bridgman seal [9] according to a design proposed by Poulter [10]. The vessel was capable of supporting pressures up to 1,500 bars, and had been built with a surrounding oil bath for temperature control.

**Figure 3:**
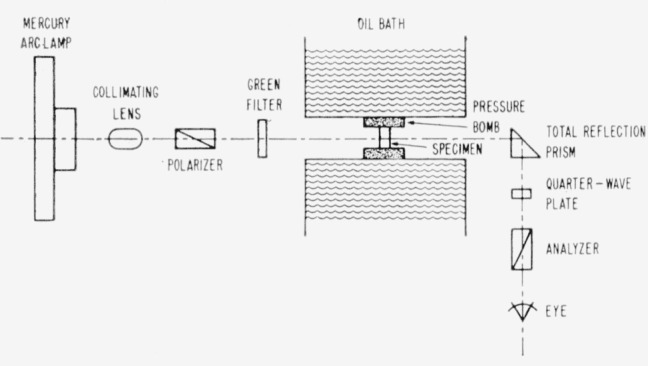
Arrangement of optical system to measure optical path-difference.

In order to observe relaxation under pressure, the polarimeter of Goranson and Adams was disassembled and remounted on a large optical bench. The pressure vessel was placed at such a position in the optical train that it enclosed the specimen. As a safety measure against possible failure of the window, a total reflection prism was placed in the optical train immediately after the specimen, so that the light beam was turned through 90°. The light beam then passed through the quarter-wave plate and analyzer which were placed at right angles to the rest of the optical train (fig. 3). With this arrangement, the reflection of the polarized light had no effect upon the measurement of phase lag in the specimen beyond changing the zero point in taking readings.

Photoelastic effects caused by hydrostatic pressure were observed in the glass windows of the vessel, and these effects increased in magnitude with increasing pressure. A black cross typical of a radial distribution of stress appeared when the windows were examined between crossed polaroids with white light. With the aid of a sensitive red tint plate, it was estimated that at a pressure of 1,000 bars, the optical path-difference varied from zero at the center of the aperture to about 50 m*μ* at the edge. This maximum was reduced to about 25 m*μ* by placing a diaphragm at the window. Using the polarimeter with monochromatic light produced an integrated effect of this variation so that it was slightly more difficult to make a photometric match at the higher pressures.

The pressure in the bomb was measured by means of a calibrated Bourdon gage to the nearest 7 bars. A mercury contact thermoregulator held the temperature in the oil bath surrounding the bomb to within ±0.02 °C.

**Figure 4:**
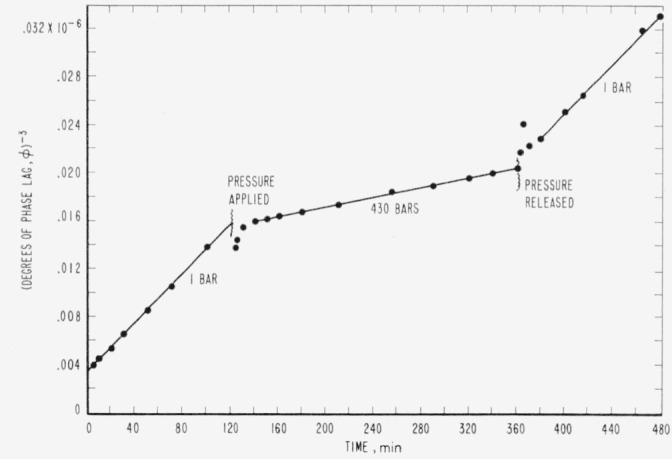
The reciprocal cubed of phase lag in allyl diglycol carbonate as a function of time, showing the effect of a hydrostatic pressure of 430 bars in comparison with that of 1 bar.

Measurements were then made of relaxation while a specimen was under hydrostatic pressure, and the results of a typical experiment are shown in figure 4. The specimen was first allowed to relax at atmospheric pressure for a period of 120 min, and then the hydrostatic pressure was applied and held for a period of 240 min.

The reciprocal cubed of phase lag was plotted against time in minutes and it can be seen from figure 4 that the fourth power relationship for relaxation (1) really holds at elevated pressure. The irregularities in the points immediately after changes in pressure are attributable to associated, adiabatic temperature changes. The slope of the straight line which represents the parameter, 3*A_p_*, was found, therefore, from direct measurements rather than by determining the slope by inference, as had been done before a pressure bomb with windows was available. The parameter, 3*A*_0_, was found from the slope of the straight line in the first, 120-min period, when the specimen was at atmospheric pressure, and the relative mobility was calculated.

**Figure 5:**
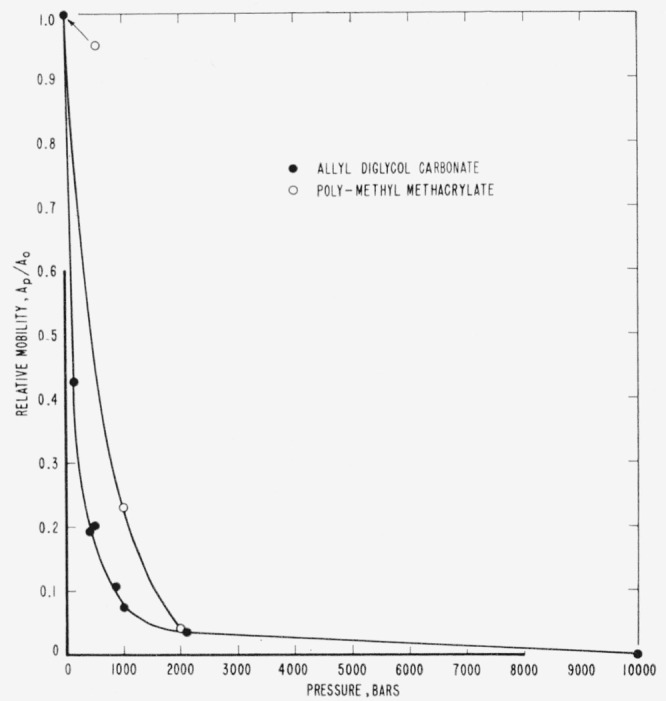
Relative mobility as a function of pressure at 24 °C.

Experiments at 24 °C were conducted on allyl diglycol carbonate at pressures of 162, 430, and 869 bars. Relative mobilities were calculated and these data were combined with data obtained by the old method of estimating the relaxation under pressure (see fig. 2). The relative mobilities versus pressure at 24 °C are shown in figure 5 for both allyl diglycol carbonate and polymethyl methacrylate.

## 8. Effect of Temperature

**Figure 6:**
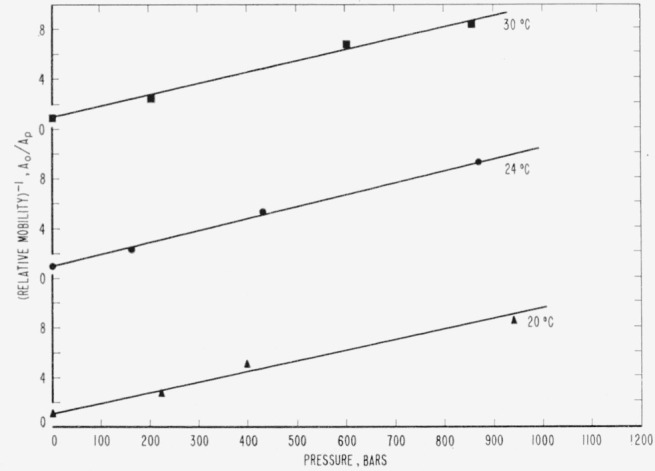
(Relative mobility)^−1^ in allyl diglycol carbonate as a function of pressure at 3 temperatures.

In order to gain some conception of the relative effects of temperature and pressure upon the relaxation of allyl diglycol carbonate, additional experiments were conducted at 20 °C and 30 °C. The observations were made, as nearly as possible, at pressures of 200, 500, and 1,000 bars, a freshly cut specimen being used each time. When relative mobilities were calculated from the data, it was found that a straight line represented the relationship 1/(relative mobility) versus pressure at a given temperature, and this is shown in figure 6. The data for all the relative mobilities resulting from change in pressure are given in table 1.

**Figure 7:**
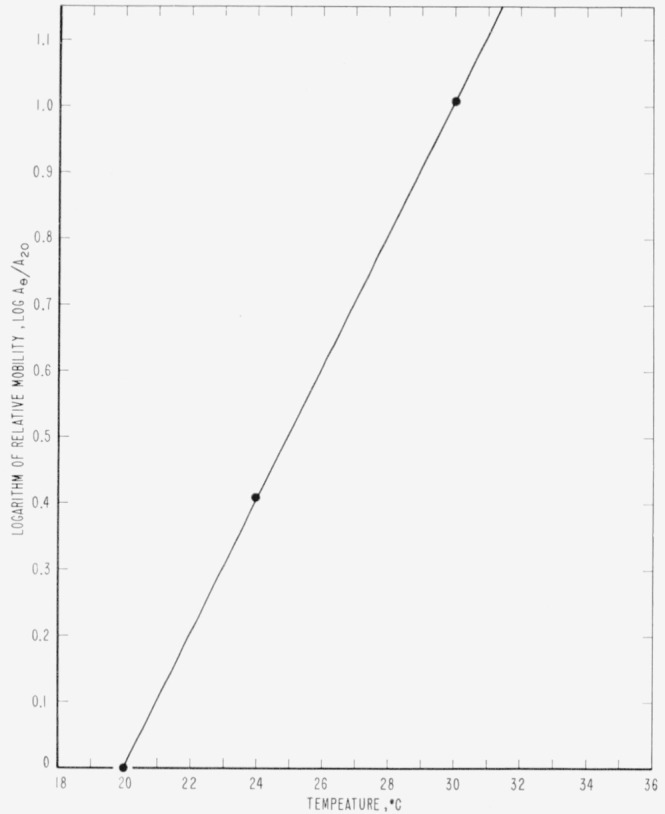
Effect of temperature upon the relative mobility of allyl diglycol carbonate at atmospheric pressure.

One experiment was conducted at atmospheric pressure to determine the value of 3 *A* for the same specimen of allyl diglycol carbonate at 20, 24, and 30 °C. Using the slope of the straight line at 20 °C as a basis, relative mobilities were determined, and they appeared to vary exponentially with temperature according to the equation

$$\mathrm{Log}\frac{A_{\theta }}{A_{20}}=0.106\;\theta -2.120$$ (3)

where *A_θ_* is the mobility at the given temperature, *θ*, and *A*_20_ is the mobility at 20 °C. This relationship is shown in figure 7, and the data are shown in table 2.

## 9. Discussion

As can be seen in figure 5, the flow in amorphous solids is very sensitive to the effect of hydrostatic pressure and even the comparatively low pressure of 162 bars is sufficient to reduce the relative mobility of allyl diglycol carbonate to less than half its value at atmospheric pressure. The effect becomes less pronounced at higher pressures, but a pressure of 10,000 bars is sufficient to inhibit the flow completely. The effect in polymethyl methacrylate is comparable, but not as great as in allyl diglycol carbonate.

At pressures below 1,000 bars a linear relationship appears to hold for 1/(relative mobility) versus pressure for allyl diglycol carbonate as shown in figure 6. At higher pressures the relationship ceases to be linear and, as the pressure increases, 1/(relative mobility) increases rapidly. Since the slopes of the straight lines in figure 6 are nearly the same, it appears that the pressure dependence of 1/(relative mobility) is not affected by temperature, at least not over the range of pressure and temperature investigated.

If this variable, 1/(relative mobility), or relative resistance to flow is considered, it is interesting to compare the results of the present investigation with those of Bridgman [9] in his work on the viscosity of liquids under pressure. Bridgman points out that the viscosity of a liquid increases with pressure at a rapidly increasing rate. He also shows that the relative change of viscosity with temperature be comes markedly greater at high pressure, whereas most temperature effects become less at high pressures. Bridgman comments on the large magnitude of the pressure effect, and the large variation from liquid to liquid, and these observations apply generally to all the liquids studied, although water is somewhat abnormal in its behavior, and shows the effect of association at low temperatures and pressures.

From Bridgman’s tabulated data it can be seen that oleic acid showed an increase of fourfold in relative viscosity upon increasing the pressure from 1 bar to 1,000 bars at 30 °C. Allyl diglycol carbonate showed a ninefold increase in relative resistance to flow for the same pressure change at the same temperature. Of all the liquids investigated by Bridgman, oleic acid showed the greatest pressure dependence of relative viscosity for pressures up to 1.000 bars. Eugenol showed the greatest pressure dependence up to 12,000 bars, and Bridgman calculated that this increase of pressure at 30 °C would increase the relative viscosity by 10^7^. Although measurements were made at a slightly lower temperature (24 °C), the relative resistance to flow of allyl diglycol carbonate became infinitely great at 10.000 bars.

## Figures and Tables

**Table 1:** Dependence of relative mobility upon pressure at constant temperature

Material	Temperature	Pressure bars	Relative mobility *A_p_/A*_0_
	***°C***		
Allyl diglycol carbonate	20	$\{\;\begin{array}{r} 1\\ 223\\ 397\\ 941 \end{array}$	1.000
			0.357
			.198
			.115
	24	$\{\begin{array}{r} 1\\ 162\\ 430\\ 500\\ 869\\ 1,000\\ 2,000\\ 10,000 \end{array}$	1.000
			0.427
			.193
			.203
			.107
			.0746
			.0364
			0.0
	30	$\{\;\begin{array}{r} 1\\ 204\\ 601\\ 855 \end{array}$	1.000
			0.405
			.145
			.117
Poly methyl methacrylate	24	$\{\;\begin{array}{r} 1\\ 1,00\\ 2,00 \end{array}$	1.000
			0.230
			.0403

**Table 2:** Dependence of relative mobility upon temperature at atmospheric pressure

Material	Pressure bars	Temperature	Relative mobility *A_θ_/A*_20_	Logarithm of relative mobility Log *A_θ_*/*A*_20_
		°*C*		
Allyl diglycol carbonate	1	$\{\;\begin{array}{c} 20\\ 24\\ 30 \end{array}$	1.0	0.0
			2.56	.409
			11.49	1.060

## Footnotes

1 Figures in brackets indicate the literature references at the end of this paper.

2 The authors are indebted to the Geophysical Laboratory of the Carnegie Institute of Washington for the loan of this instrument.

3 This pressure vessel was kindly loaned by Dr. R. E. Gibson, Director of the Applied Physics Laboratory of the Johns Hopkins University.
